# Accuracy of Physical Examination to Detect Synovial and Extra-Synovial Pathologies in Psoriatic Arthritis in Comparison to Ultrasonography

**DOI:** 10.3390/jcm9092929

**Published:** 2020-09-10

**Authors:** Ummugulsum Gazel, Dilek Solmaz, Gizem Ayan, Catherine Ivory, Jacob Karsh, Sibel Zehra Aydin

**Affiliations:** 1 Rheumatology, University of Ottawa Faculty of Medicine, Ottawa, ON K1H 7W9, Canada; drgizemayan@gmail.com (G.A.); civory@toh.ca (C.I.); JKARSH@toh.ca (J.K.); saydin@toh.ca (S.Z.A.); 2 Ottawa Hospital Research Institute (OHRI), Ottawa, ON K1H 7W9, Canada; 3 Department of Internal Medicine, Division of Rheumatology, Izmir Katip Celebi University, Cigli, Izmir 35620, Turkey; d.solmaz2012@gmail.com

**Keywords:** psoriatic arthritis, ultrasonography, imaging

## Abstract

We aimed to explore the accuracy of physical examination (PE) to detect the synovial and extra-synovial pathologies in psoriatic arthritis (PsA) in comparison to ultrasonography (US). Twenty-nine PsA patients with hand pain were included in the study. A detailed PE of the hands was performed and US scans were performed for the joints, extensor and flexor tendons, and entheses of the second to fifth fingers of both hands. The agreement between PE and US findings was calculated. The strongest agreement for the joints was between “swollen joints” and power Doppler (PD) signals in the metacarpophalangeal (MCP) joints and grey scale synovitis in the proximal interphalangeal (PIP) joints. The agreement of tender entheses on PE and inflammation on US (hypoechogenicity, thickening, and/or PD signals) was poor for both extensor (Kappa = −0.027, Prevalence Adjusted and Bias Adjusted Kappa (PABAK) = 0.344) and flexor compartments (Kappa = 0.039, PABAK = 0.569). Similar to enthesitis, comparison of any PE and US findings showed a poor agreement at the extensor and flexor tendon regions (extensor: Kappa = 0.123, PABAK = 0.448, and flexor: Kappa = 0.171, PABAK = 0.431). Our study showed that there was a poor to fair agreement of PE and US findings of hands. US can add value when determining the source of pain in PsA in the small joints.

## 1. Introduction

Psoriatic arthritis (PsA) is a chronic heterogeneous inflammatory disease with articular and extra-articular manifestations [1]. Imaging studies on the hands showed that extra-synovial features such as flexor tendon enthesitis and peritendinous edema are exclusively seen in PsA [2]. Even in the absence of any significant joint inflammation, the extensor tendon and paratenon, the flexor tendons and tendon sheaths, as well as their insertions, the skin, and even the pulleys can be inflamed in PsA, which may all contribute to various clinical presentations [3,4,5]. Physical examination (PE) to differentiate these abnormalities (synovial vs. extra-synovial) can be challenging due to the proximity of these structures. Musculoskeletal ultrasonography (US) was demonstrated to be useful in the assessment of the inflammatory process at the level of synovial and extra synovial tissues [6]. The importance of the identification of structures involved in PsA lies in the efficacy of treatments that can be different for different disease manifestations [7]. All randomized controlled trials (RCTs) in PsA have mainly focused on the effects of treatments on synovial disease based on PE. None used imaging to differentiate the involvement of different structures or the responsiveness of different pathological lesions in small joints, such as hands and feet, to various treatments [8]. Although a few studies explored the specificity of extra-articular features in PsA, none of the studies looked at the contribution of these pathologies to pain or the accuracy of PE to distinguish these lesions. Our aim was to explore the accuracy of PE to detect the involvement of synovial and extra-synovial structures of hands in PsA in comparison to ultrasonography as the gold standard, and to determine how often these extra-synovial pathologies lead to symptoms.

## 2. Method

### 2.1. Patient Selection

This was a prospective data collection with cross-sectional analysis performed at the Ottawa Hospital, Arthritis Center, University of Ottawa (Ottawa, ON, Canada). Adult patients (≥18 years old) who fulfilled the Classification Criteria for the Study of Psoriatic Arthritis (CASPAR) criteria for PsA and pain in at least one hand at the current visit were recruited. Exclusion criteria included other concomitant diseases affecting the hands clinically (e.g., osteoarthritis or gout) or history of hand fracture or surgery.

Data on demographics, clinical characteristics, and medication history were collected. Health Assessment Questionnaire (HAQ), patient’s global assessment of disease activity (Pt-GA), patient’s pain assessment (Pt-pain), Body Surface Area (BSA), physician global assessment (PGA), and Patient Acceptable Symptom State (PASS) were also recorded. Laboratory findings of C-reactive protein (CRP), erythrocyte sedimentation rate (ESR), and human leukocyte antigen (HLA)-B27 were recorded, whenever available [9].

A detailed PE was performed by a single experienced investigator (J.K.) to differentiate pain due to joint, tendon, and entheseal disease. Palpation was done at the level of the metacarpophalangeal (MCP), proximal interphalangeal (PIP), and distal interphalangeal (DIP) joints to detect fluid or tenderness. Each joint was defined as “swollen” and/or “tender” by PE. Additional analysis was performed to combine the PE findings of tenderness and swelling, or having either one of them. Tenderness of the entheses at the insertion of the flexor and extensor tendons at the region of the DIP joints and insertion at the nailbeds was recorded. The flexor and extensor tendons distal to the MCP joints were assessed by palpation, passive, and resisted movements, separately.

### 2.2. Ultrasonography Protocol

All patients were assessed by US at the end of their clinical visits. All scans were performed using a MyLab-ClassC (Esaote Biomedica, Genoa, Italy), equipped with a broadband 6–18 MHz linear probe. The US scans were performed by an investigator blinded to the clinical assessment, on dorsal and palmar views, in neutral position. Power Doppler (PD) settings were standardized with a pulse repetition frequency of 500 Hz and low wall filter and gain adjusted until the background signal was removed. Eight digits (2nd–5th digits, bilaterally) were scanned per patient. A total of 696 joints (MCP, PIP, and DIP joints), 464 extensor and flexor tendons, and 696 entheses (insertion of the central band of the extensor tendon to the basis of the middle phalanx, lateral band insertion to the distal phalanx, and the deep flexor tendon insertion to the distal phalanx) were scanned.

Grey scale (GS) synovitis and PD signals within the joints were defined according to the definitions developed by the Outcome Measures in Rheumatoid Arthritis in Clinical Trials (OMERACT) US taskforce [10]. A semi-quantitative scoring system was used for grading. GS synovitis was scored between 0 and 3: 0, none; 1, mild; 2, moderate; and 3, marked synovial thickening. For scoring the PD signal, the following was used: score 0, no PD signal; score 1, one or two vessels (including one confluent vessel) for small joints and two or three signals for large joints (including two confluent signals); score 2, a PD signal of greater than score 1, but less than 50% of the area; score 3, a PD signal covering >50% of the GS synovitis.

**Figure 1:**
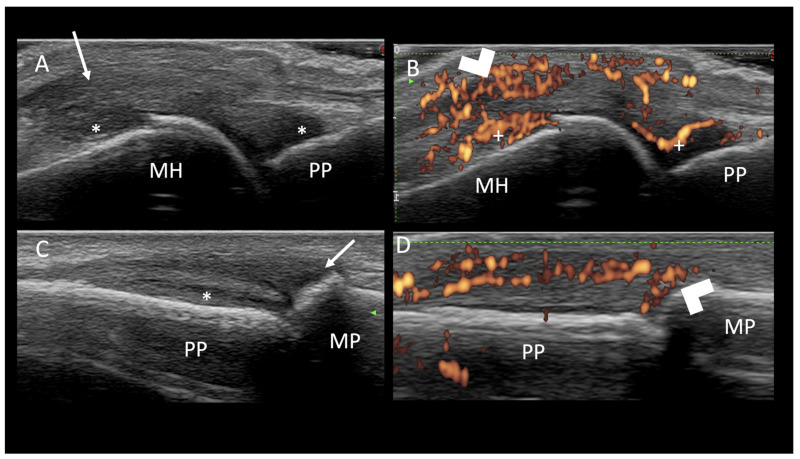
Longitudinal views of the metacarpophalangeal (**A**,**B**) and proximal interphalangeal joints (**C**,**D**) on ultrasound. (**A**) Synovial proliferation (∗) within the metacarpophalangeal joint and thickening, hypoechogenicity, and loss of fibrillary echotexture of the extensor tendon (arrow). (**B**) Intratendineous power Doppler signals (arrow head) in addition to the intrasynovial Doppler signals within the metacarpophalangeal joint (+). (**C**) Synovial proliferation of the proximal interphalangeal joint (∗) and thickening, hypoechogenicity, and loss of fibrillary echotexture of the extensor tendon insertion into the basis of the middle phalanx- enthesitis (arrow). (**D**) Entheseal power Doppler signals (arrow head).

For the tendons, the following US findings were scored as present or absent: for the extensor tendons, hypoechogenicity, thickening, PD signals, and paratenonitis; for the flexor tendons, hypoechogenicity, thickening, PD signals, and tenosynovitis in GS and PD positivity with the tendon sheath. The entheses were investigated for features of inflammation (hypoechogenicity, thickening, and PD signal) as present or absent. Ultrasonography findings are described in Figure 1.

### 2.3. Statistical Analysis

Descriptive statistics are reported as median (IQR) or mean (SD) for continuous variables (depending on the distribution) or as frequencies (percentages) for categorical variables. The accuracy of PE to detect synovial and extra-synovial pathologies by taking US as the gold standard was compared using a 2 × 2 table, separately for the joints, tendons, and entheses. For the joints, analysis used the presence or absence of GS synovitis and PD signals on US, as well as using the cut-off >1 for both. The level of agreement between PE and US was evaluated using Kappa statistics: 0–0.2, poor; 0.21–0.4, fair; 0.41–0.6, moderate; 0.61–0.8, substantial; and 0.81–1, almost perfect [11]. Since Kappa is highly dependent on the prevalence of the lesion, the Prevalence Adjusted and Bias Adjusted Kappa (PABAK) were also calculated [12,13]. The agreements were assessed for MCP, PIP, and DIP joints separately. For joints, PE was categorized as “tender”, “swollen”, “tender and swollen”, or “tender or swollen joints” and compared to PD or GS in the US. For the entheses, tenderness was compared to any US findings (hypoechogenicity and/or inflammation and PD) similar to the tendons. Stata (Stata Corp LLC, Stata Statistical Software: Release 16. College Station, TX, USA) V16 was used for analysis.

### 2.4. Ethical Approval

All procedures performed in this study were in accordance with the ethical standards of the institutional research committee (Ottawa Health Science Network Research Ethics Board, Ottawa (20180470) and with the 1964 Helsinki Declaration and its later amendments or comparable ethical standards. Informed consent was obtained from all patients prior to data collection.

## 3. Results

### 3.1. Patient Characteristics

Fifteen of the 29 patients were male and their mean age was 54.8 (8.4) years. Mean (SD) PsA duration was 15.3 (10) years and around half of the patients were never smokers (*n* = 17, 55.2%). Mean (SD) BSA was 2.1 (2.09) and nail involvement rate was 77% (*n* = 22). Approximately one-third of patients had (current and/or past) had dactylitis (*n* = 9), enthesitis (*n* = 10), or deformities (*n* = 9), and 15% of patients had uveitis (*n* = 4). Polyarticular and oligoarticular phenotype rates were 83% and 3.5%, respectively, and 10% of patients had axial disease according to the clinician. None of the patients had monoarticular involvement and DIP joint arthritis was found in 14%. For disease activity, the mean (SD) BSA was 3.1 (2.6) and pain was 5.5 (2.3). PASS was reported in 58.6% of patients and mean (SD) HAQ was 0.67 (0.58).

### 3.2. Physical Examination Findings

PE findings for 232 digits are summarized in Table 1. Tender joint count (TJC) was detected the most often in MCP joints (*n* = 122 (52.5%)) in 27 patients (93%), and swollen joint count (SJC) was highest in PIP joints (*n* = 79 (34%)) in 23 patients (79%).

For the entheses, tenderness was detected in 58/232 (25%) of the extensor site, in 21/29 (72%) of patients, and 46/232 (20%) of the flexor site, in 18/29 (62%) of patients. For the tendons, any PE findings (tenderness on palpation, passive or resisted movements) were found in 64/232 (27.6%) of the extensor tendons in 19/29 (65.5%) patients, and 68/232 (29%) of the flexor tendons in 22/29 (79%) patients (Table 1).

Overall, among the 232 digits examined, 178 were found to be tender on PE. Within tender digits, the MCP joint tenderness was the leading feature (*n* = 122/178 (69%)) of PE, followed by PIP joint tenderness (*n* = 100/178 (56%)). The least frequent PE finding in tender digits was extensor tendon swelling (*n* = 6/178 (3.4%)).

### 3.3. Ultrasonography Findings

GS synovitis was found highest in MCP joints (*n* = 89/232, 38.4% of the joints in *n* = 24/29, (83% of the patients)). PD positivity was similar in MCP (15/232, 6.5% of the joints; 7/29, 24% of the patients) and PIP joints (16/232, 7% of the joints; 8/29, 27.5% of the patients). Overall, DIP joints had low GS and PD positivity rates (1.3% and 0.9%, respectively).

The extensor and flexor compartments were similarly affected and thickening was the most common finding in extensor tendons: *n* = 24/232, 10% of tendons; 10/29, 34.4% of the patients; and flexor tendons: *n* = 27/232, 11.6% of tendons; 8/29, 27.5% of the patients.

Hypo-echogenicity and thickening were found more frequently in the extensor tendon middle phalanx insertion site (*n* = 23/232 (10%) in 9 (31%) patients and *n* = 39/232 (16.8%) in 16 (20.6%) patients, respectively). PD positivity was detected only in the minority for all entheseal sites (Table 2).

### 3.4. Agreement of Physical Examination and Ultrasonography

For the MCP joints, the strongest agreement was between swollen joints on PE and PD signals on the US (Kappa = 0.240, PABAK = 0.827). In the PIP joints, the strongest agreement was between tender and swollen joints on PE and GS synovitis on the US (Kappa = 0.330, PABAK = 0.551). For DIP joints, all the agreement results showed poor Kappa values (Table 3). When using the cutoff of >1 for GS synovitis and PD signals, agreements for the joints did not improve.

Comparison of any PE and US findings showed a poor agreement for the extensor and flexor tendon regions of the hands (Kappa = 0.123, PABAK = 0.448, and Kappa = 0.171, PABAK = 0.431, respectively).

For the entheses, tenderness on PE and any US finding of inflammation showed a poor agreement for both extensor (Kappa = −0.027, PABAK = 0.344) and flexor sites (Kappa = 0.039, PABAK = 0.569) (Table 3).

### 3.5. Distribution of Ultrasonography Findings According to Tenderness

The US findings of all tender and non-tender digits are summarized in Table 4. Within tender digits, the MCP joint GS synovitis was found in 73/178 (41%), and 13/178 (7.3%) of the digits had PD signals. For PIP joints, GS synovitis and PD signals were present in 32/178 (18%) and 15/178 (8.4%) of tender digits, respectively. GS synovitis and PD signals were found in three (1.7%) and two (1.1%) in DIP joints among tender digits. Extensor and flexor tendon US positivity were 16/178 (9%) and 24/178 (13.4%) within tender digits, respectively. In extensor middle and distal phalanx entheses, 32/178 (18%) and 30/178 (17%) of tender digits had US positivity, respectively.

## 4. Discussion

Our study showed that there is a poor to fair agreement of PE and US findings for the joints, tendons, and entheses of hands, which may all be involved and cause pain in PsA. Various therapeutic choices may eventually be found to have different effects on the inflammation of these structures; therefore, US assessment of the hands may guide physicians to localize the source of pain better than the PE and thus suggest therapeutic options.

There is evidence on the link between PE and US to assess synovitis in small joints [14]. Turner et al. investigated the US findings on the foot in PsA and found that higher body mass index (BMI), female sex, subluxation, and erosions were independent predictors of metatarsophalangeal (MTP) joint pain in PsA patients in addition to US-detected synovitis [15]. They clinically found 129 (32%) painful and 10 (3%) swollen MTP joints, while US-synovitis and PD positivity was less often, detected in 47 (14%), and 6 (2%) in MTP joints. In another study by Naranje et al., PD signals were detected in only a few joints in the hands that were clinically tender or swollen; however, they found significant correlation between US measures (GS joint count, GS joint score, and PD joint score) and Disease Activity Score 28 scores [16]. They found strong correlations between TJC and all US measures, but not the SJCs. Although these two studies demonstrated the prevalence of joint findings according to PE, neither investigated tender and entheseal lesions on US or PE. However PsA is a heterogeneous disease that is not limited to the joints and the involvement of various structures can lead to pain. For entheses, studies in the literature focused mostly on lower limb entheses and knowledge to compare PE vs. US is limited for extra-synovial features of the hands [14]. Our study demonstrated that tendon and entheseal lesions are found in 9% to 18% of the tender digits, which represents the heterogeneity of PsA and how often the extraarticular findings contribute to pain. This suggests limiting the assessment to the joints underestimates the extent of inflammation.

Previous reports demonstrated that MCP and PIP joints were similarly affected in early PsA, which was also observed in our study on the PE, in a more established PsA population [17,18,19]. However, there are differences in terms of the US findings where MCP joints were more frequently involved on the US compared to the PIP joints, therefore using US also allows the accurate assessment of joint disease, in addition to its benefits to evaluate the extraarticular structures.

The literature suggests that US examination allows detection of extra-synovial pathologies that are more specific to PsA and improves the diagnosis of the disease. Recently, Macía-Villa et al. identified that in MCP joints, peritenon extensor tendon inflammation and intraarticular synovitis can cause clinically prominent swelling at the same rate [18]. In two studies by Zabotti et al., extra-synovial features of hands, such as peritendon extensor tendon inflammation, central slip enthesitis, and soft tissue edema, were found more commonly in PsA than rheumatoid arthritis (RA) [19,20]. Flexor tendon pulleys, which are also extra-synovial structures that can be involved in PsA, were found to be thickened in PsA subjects in comparison with subjects with RA, psoriasis patients, and healthy controls [4]. Due to its proximity to the DIP joint and extensor enthesis, the nail has been widely investigated with US as another extra-synovial structure in PsA patients. In a systematic literature review, the nail plate change rate was reported with a variance of ˂10% to over 97% in PsA and psoriasis [21]. Additionally, US-detected soft tissue edema was suggested to be relevant for early diagnosis of dactylitis and PsA [22]. In our study, the agreements of the US and PE were poor for the entheses and the tendons, which suggested that in order to fully understand the structures being involved in PsA, US would add value to PE, and implementing US in the standard of care as well as research would improve the understanding of PsA pathogenesis.

As the frequency of some lesions was fairly low, we also calculated PABAK in addition to kappa values. PABAK is a hypothetical estimation of the agreement as if there had been a sufficient sample size [23,24]. Our study showed that PABAK values were greater than kappa values for the agreement of MCP joint swollen and PD positivity, DIP joint swollen and GS/PD positivity, and extensor and flexor tendon US and PE findings, which may be due to the lower prevalence of these lesions. Agreement in these regions might be improved with a larger sample size.

Our study has limitations. We used only a single, albeit experienced, examiner to perform the PE so we cannot address the variability of findings that may occur using other examiners of more or less experience. Similarly, the variability of US evaluations by numerous examiners is also unknown. We studied patients with a long duration of disease and on numerous medications at a single point in time. We cannot address the variability of the findings over time or the ability of findings to change either spontaneously or through therapeutic intervention. Chronicity of inflammation and the older age of patients might have impacted our findings. Although patients with clear OA of the hands were excluded, there may still be symptoms due to early OA, which might have affected the PE but not US findings. Thereby, the US assessment in this patient population may have an even higher value as it can be challenging to differentiate OA and PsA pain and US may provide objective measures. The lack of OA patient control group can be considered a limitation in detecting differences in the clinical and ultrasonographic findings of two diseases. Whether the agreement would be higher in a younger population with earlier disease requires further testing. Considering the feasibility, our US assessment was limited to certain anatomical sites and did not include some structures such as the collateral ligaments and proximal insertion of the flexor tendon. However, in our experience, these structures are rarely affected on US in PsA patients. We therefore did not expect these to have a major influence on our results. Additionally, the reason for weak agreement between PE and US in our study may be associated with the lower prevalence of the findings, as explained above. Our sample of 29 patients may be considered small, but the study assessed hundreds of articular and extra-articular structures, providing robust statistical validity to the findings.

In summary, hand pain in PsA may be associated with a variety of anatomical structures, as allocated among inflammatory and non-inflammatory causes. As assessed by US, the responsiveness of inflammation in these structures to various treatments may represent important outcome measures in future therapeutic trials in psoriatic disease and may provide a guide to personalized medicine in psoriatic patients.

## Figures and Tables

**Table 1:** Physical examination findings.

Physical Examination of the Joints and Entheses				
	**Tender**	**Swollen**	**Tender and Swollen**	**Tender or Swollen**
**MCPj, *n* (%)**	122 (52.5)	13 (5.6)	12 (5.2)	123 (53)
**PIPj, *n* (%)**	100 (43.1)	79 (34)	61 (26.3)	117 (50.4)
**DIPj, *n* (%)**	97 (42)	19 (8.2)	13 (5.6)	100 (43)
**Extensor Entheses, *n* (%)**	58 (25)			
**Flexor Entheses, *n* (%)**	46 (20)			
**Physical examination of Tendons**				
	**Tender against resistance**	**Tender against palpation**	**Swollen**	**Any finding**
**Extensor, *n* (%)**	34 (14.7)	48 (20.7)	13 (5.6)	64 (27.6)
**Flexor, *n* (%)**	31 (13.4)	47 (20.3)	21 (9)	68 (29)

**Table 2:** Ultrasonography findings.

Articular US Findings											
	Grey scale positivity						Power Doppler positivity				
	Any			≥2			Any			≥2	
**MCPj, *n* (%)**	89 (38.4)			24 (10.3)			15 (6.5)			10 (4.3)	
**PIPj, *n* (%)**	35 (15)			15 (6.5)			16 (7)			7 (3)	
**DIPj, *n* (%)**	3 (1.3)			1 (0.4)			2 (0.9)			0	
**Tendon US Findings**											
	**Hypo-echogenicity**		**Thickening**		**Doppler**		**Paratenonitis**	**Tenosynovitis Gray Scale**			**Tenosynovitis Doppler**
**Extensor**	19 (8)		24 (10)		13 (5.6)		3 (1.3)				
**Flexor**	18 (7.8)		27 (11.6)		14 (6)			12 (5)			6 (2.6)
**Entheseal US Findings**											
		**Hypo-echogenicity**				**Thickening**			**Doppler**		
**Extensor tendon Middle phalanx insertion**		23 (10)				39 (16.8)			3 (1.3)		
**Extensor tendon Distal phalanx insertion**		8 (3.4)				31 (13.4)			7 (3)		
**Flexor tendon Distal phalanx insertion**		6 (2.6)				9 (4)			3 (1.3)		

Numbers are given as *n* (%) per joints/entheses/tendons.

**Table 3:** Agreement of physical examination and ultrasonography in joints, enthesis, and tendons.

			Power Doppler		Kappa/PABAK		Grey Scale		Kappa/PABAK
			Absent	Present			Absent	Present	
**MCPj**	**Tender joints, *n***	**Absent**	108	2	0.084/0.043		73	37	0.089/0.077
		**Present**	109	13			70	52	
	**Swollen joints, *n***	**Absent**	208	11	0.240/0.827		139	80	0.087/0.275
		**Present**	9	4			4	9	
	**Tender AND Swollen joints, *n***	**Absent**	208	12	0.175/0.819		139	81	0.074/0.267
		**Present**	9	3			4	8	
	**Tender OR Swollen joints, *n***	**Absent**	108	1	0.099/0.051		73	36	0.098/0.086
		**Present**	109	14			70	53	
**PIPj**	**Tender joints, *n***	**Absent**	128	4	0.099/0.206		123	9	0.208/0.284
		**Present**	88	12			74	26	
	**Swollen joints, *n***	**Absent**	152	1	0.227/0.439		144	9	0.312/0.465
		**Present**	64	15			53	26	
	**Tender AND Swollen joints, *n***	**Absent**	167	4	0.227/0.543		158	13	0.330/0.551
		**Present**	49	12			39	22	
	**Tender OR Swollen joints, *n***	**Absent**	114	1	0.119/0.112		109	6	0.195/0.189
		**Present**	102	15			88	29	
**DIPj**	**Tender joints, *n***	**Absent**	134	1	0.003/0.163		133	2	−0.005/0.155
		**Present**	96	1			96	1	
	**Swollen joints, *n***	**Absent**	211	2	−0.016/0.819		210	3	−0.023/0.810
		**Present**	19	0			19	0	
	**Tender AND Swollen joints, *n***	**Absent**	217	2	−0.015/0.870		216	3	−0.021/0.862
		**Present**	13	0			13	0	
	**Tender OR Swollen joints, *n***	**Absent**	131	1	0.003/0.137		130	2	−0.006/0.129
		**Present**	99	1			99	1	
**Tenderness on PE**		**Any inflammation on US within the enthesis, *n***							**Kappa/PABAK**
		**Absent**				**Present**			
EE *	**Absent**	149				25			−0.027/0.344
	**Present**	51				7			
**FE**	**Absent**	179				7			0.039/0.569
	**Present**	43				3			
**Tenderness on PE**		**Any inflammation on US within the tendons, *n***							**Kappa/PABAK**
		**Absent**				**Present**			
**ET**	**Absent**	158				10			0.123/0.448
	**Present**	54				10			
**FT**	**Absent**	151				13			0.171/0.431
	**Present**	53				15			

* Results are only given for the insertions of the tendons to the basis of distal phalanx.

**Table 4:** Distribution of ultrasonography findings according to tenderness.

US Findings		Tenderness of Digits *, *n*	
		Present (*n* = 178) *n* (%)	Absent *(n* = 54) *n* (%)
**MCPj GS synovitis**	**Present**	73 (41)	16 (30)
	**Absent**	105 (59)	38 (70)
**MCPj Power Doppler**	**Present**	13 (7.3)	2 (3.7)
	**Absent**	165 (93)	52 (96.3)
**PIPj GS synovitis**	**Present**	32 (18)	3 (5.5)
	**Absent**	146 (82)	51 (94.5)
**PIPj Power Doppler**	**Present**	15 (8.4)	1 (1.8)
	**Absent**	163 (91.6)	53 (98.2)
**DIPj GS synovitis**	**Present**	3 (1.7)	0
	**Absent**	175 (98.3)	54 (100)
**DIPj Power Doppler**	**Present**	2 (1.1)	0
	**Absent**	176 (99)	54 (100)
**Extensor Tendon**	**Present**	16 (9)	4 (7.5)
	**Absent**	162 (91)	50 (92.5)
**Flexor Tendon**	**Present**	24 (13.4)	4 (7.5)
	**Absent**	154 (86.6)	50 (92.5)
**Extensor tendon** **Middle phalanx insertion**	**Present**	32 (18)	9 (16.6)
	**Absent**	146 (82)	45 (83.4)
**Extensor tendon** **Distal phalanx insertion**	**Present**	30 (17)	2 (3.7)
	**Absent**	148 (83)	52 (96.3)
**Flexor tendon** **Distal phalanx insertion**	**Present**	10 (5.6)	0
	**Absent**	168 (94.4)	54 (100)

* Tenderness due to any cause joints, tendons or entheses on exam in that digit.

## Abbreviations

DIPj	Distal interphalangeal joint
EE	Extensor enthesis
ET	Extensor tendon
FF	Flexor enthesis
FT	Flexor tendon
GS	Grey scale
MCPj	Metacarpophalangeal joint
MH	Metacarpal head
MP	Middle phalanx
PABAK	Prevalence adjusted and bias-adjusted kappa
PIPj	Proximal interphalangeal joint
PP	Proximal phalanx
US	Ultrasonography
